# Zinc-Containing Hydroxyapatite Enhances Cold-Light-Activated Tooth Bleaching Treatment In Vitro

**DOI:** 10.1155/2017/6261248

**Published:** 2017-10-12

**Authors:** Yi Li, Xinchang Shi, Wei Li

**Affiliations:** ^1^State Key Laboratory of Oral Diseases, Chengdu 610041, China; ^2^School of Basic Medical Science, Zhengzhou University, Zhengzhou 450001, China; ^3^Bybo Dental Group, Zhengzhou 450000, China

## Abstract

Cold-light bleaching treatment has grown to be a popular tooth whitening procedure in recent years, but its side effect of dental enamel demineralization is a widespread problem. The aim of this study was to synthesize zinc-substituted hydroxyapatite as an effective biomaterial to inhibit demineralization or increase remineralization. We synthesized zinc-substituted hydroxyapatite containing different zinc concentrations and analysed the product using X-ray diffraction (XRD), Fourier transform infrared (FTIR) spectroscopy, and energy dispersive spectrometer (EDS). The biological assessment of Zn-HA was conducted by CCK-8 assay and bacterial inhibition tests. pH cycling was performed to estimate the effect of Zn-HA on the enamel surface after cold-light bleaching treatment. The XRD, FTIR, and EDS results illustrated that zinc ions and hydroxyapatite combined in two forms: (1) Zn^2+^ absorbed on the surface of HA crystal and (2) Zn^2+^ incorporated into the lattice of HA. The results indicated that 2% Zn-HA, 4% Zn-HA, and 8% Zn-HA effectively inhibited the growth of bacteria yet showed poor biocompatibility, whereas 1% Zn-HA positively affected osteoblast proliferation. The XRD and scanning electron microscopy (SEM) results showed that the use of Zn-HA in pH cycling is obviously beneficial for enamel remineralization. Zinc-substituted hydroxyapatite could be a promising biomaterial for use in cold-light bleaching to prevent enamel demineralization.

## 1. Introduction

Tooth whitening bleaching has shown extraordinary advances in modern aesthetic dentistry because it is considered a highly effective, biologically safe treatment for removing intrinsic and extrinsic stains. The current bleaching mechanism is based on the hydrogen peroxide oxidation of the pigment on the tooth surface or in a pulp chamber [1]. In the tooth whitening process, the active agent hydrogen peroxide penetrates into the tooth enamel, where it can dissociate into water, oxygen, and some species of free radicals that interact with chromophore molecules and oxidize the pigment stains.

Some studies have confirmed that hydrogen peroxide or carbamide peroxide bleaching solutions are effective in whitening discoloured teeth [2]. It is generally acknowledged that whitening bleaching is indeed a safe procedure, although its clinical side effects, such as postoperative tooth sensitivity after the treatment, should not be ignored. Some researchers have reported other side effects such as calcium loss, changes in chemical composition, alterations of surface morphology, and decreases in hardness and fracture resistance of enamel from using different methodologies [3, 4]. The main causes of the demineralization phenomenon are the oxidative effects, the composition of the agents, and the low pH gel systems that are necessary for the delivery of effective peroxide bleaching.

It is well known that synthetic hydroxyapatite (HA) is a biocompatible and bioactive synthetic material that has a considerable effect on Osteogenesis. Scientists have tried to use it in tooth bleaching paste containing concentrated H_3_PO_4_ and hydrogen peroxide to minimize the risk of demineralization [5]. However, the antibacterial property of HA is less obvious, and it could affect the remineralization process since the acidic environment causing the demineralization is produced by specific types of bacteria. It has been reported that zinc inhibits dental plaque formation in vivo due to its antibacterial effect [6]. Considering plaque removal can prevent enamel demineralization, we assume that hydroxyapatite with zinc ions would weaken the demineralization of enamel.

The objective of this article is to prepare a biomaterial mainly consisting of HA with certain zinc ions and study its phase composition, antibacterial properties, and biocompatibility. Moreover we tried to identify that the addition of zinc to the HA structure could cause cold-light bleaching-treated enamel remineralization.

## 2. Materials and Methods

### 2.1. The Synthesis of Zinc-Substituted Hydroxyapatite

Zinc-substituted hydroxyapatite was synthesized using a wet coprecipitation method, assuming that Zn would substitute for the Ca site. Zinc nitrate hexahydrate [Zn(NO_3_)_2_·7H_2_O] was used as the source for Zn, with Ca(NO_3_)_2_·4H_2_O and P_2_O_5_ dissolved into anhydrous ethanol as Ca and P precursors [7]. Briefly, 50 ml of 2 M Ca(NO_3_)_2_ solution was mixed with 2 M Zn(NO_3_)_2_ solution. Then, 50 ml of 1.2 M P_2_O_5_ solution was added to the mixture during stirring. The Ca/P ratio was maintained at 1.67, and the Zn/Ca ratio was adjusted to 0%, 1%, 2%, and 4% by adding different volumes of Zn(NO_3_)_2_ solution (0 ml, 0.5 ml, 1 ml, and 2 ml). The sol was aged at 80°C for 3 h, and pH was adjusted to approximately 6-7 using ammonium solution prior to calcination at 650°C for 2 h. We obtained 5 groups of zinc-substituted hydroxyapatite powders with Zn/(Ca + Zn) ratios of 0, 1%, 2%, 4%, and 8%; these 5 groups of zinc-containing HA were identified as group 0 Zn-HA, group 1% Zn-HA, group 2% Zn-HA, group 4% Zn-HA, and group 8% Zn-HA, respectively.

### 2.2. Characterization of the Precipitate

The phase composition of the Zn-HA was characterized by X-ray diffraction (XRD) (X'Pert PRO MPD, PANalytical Ltd., Almelo, Netherlands). The diffraction pattern was analysed over a 2*θ* range of 20°–70°, using CuKa radiation (*λ* = 1.54056 Å). A 40 kV voltage was applied to the X-ray tube, while the current intensity was 40 mA. The XRD data were then analysed using MDI Jade 5.0 and X'Pert HighScore 1.0 (Philips, Netherlands). The chemical characterization of Zn-HA was performed by Fourier transformed infrared spectroscopy (FTIR) (Nicolet 5700, Thermo Electron, USA) spectrophotometer in the region of 400–4,000 cm^−1^ on powder samples pelleted in a KBr matrix with spectral resolution of 4 cm^−1^. The chemical composition analysis of Zn-HA was performed by energy dispersive spectrometer (EDS) (Inca Penta FETX3, Oxford, UK) with MgKa radiation (1,253.6 eV) operating at 100 W.

### 2.3. Biological Assessment of Zn-HA

#### 2.3.1. Cell Proliferation

MC3T3-E1 cells (applied by State Key Laboratory of Oral Diseases) were cultured in DMEM culture medium (HyClone, USA) supplemented with 10% (v/v) FBS (HyClone, USA) and 1% penicillin-streptomycin solution at 37°C in a humidified 5% CO_2_ atmosphere incubator (Binder, Germany). To evaluate the biocompatibility property of Zn-HA, MC3T3-E1 cells were then seeded in 96-well plates at the density of 2 × 10^4^ cells/cm^2^ in Zn-HA leaching liquor. The leaching solution is prepared as follows: Zn-HA powder prepared above was sterilized under ultraviolet radiation, soaked in DMEM supplemented with 10% Foetal Bovine Serum (HyClone, USA) at a rate of 0.2 g/ml according to ISO/EN 10993-5 standards and incubated at 37°C for 48 h. After 1, 3, and 7 days of cultivation, the viability of the cells was determined by the Cell Counting Kit-8 (CCK-8) assay. Briefly, 10 *μ*l of CCK-8 solution (Dojindo, Japan) was added to each well prior to incubation for 1 h at 37°C. The optical density of each well was measured at 450 nm using a spectrophotometer Enzyme Immunoassay Analyzer (Thermo Fisher Scientific, Inc., Waltham, MA, USA) to indicate cell viability. All experiments were triplicate.

#### 2.3.2. Cell Morphology

MC3T3-E1 cells were seeded in 24-well plates at the density of 2 × 10^4^ cells/cm^2^ with the Zn-HA leaching liquor as above. After 24 h incubation, the cells were washed twice with PBS, then fixed using 2.5% glutaraldehyde (Sigma, USA) at 4°C for 1 h and rinsed three times with PBS to eliminate residual glutaraldehyde. Subsequently, the cells were dehydrated in a graded series of ethanol (30%, 50%, 70%, 90%, and 100%) for 30 min. The MC3T3-E1 cell morphology was observed under a scanning electron microscope (Inspect F, FEI Ltd., Netherlands).

#### 2.3.3. Bacterial Inhibition Test

The bacterial inhibition test was performed using a* Streptococcus mutans* strain, a Lactobacillaceae strain, and a* Streptococcus sobrinus* strain (applied by State Key Laboratory of Oral Diseases). The bacteria were stored in glycerol stock at −20°C and revived when required. For revival bacteria were incubated with TPY medium suspension at 37°C for 24 h in a shaking incubator (180 rpm) before the bacterium solution was mixed with Zn-HA leaching liquor at a ratio of 0.2 g/ml filtrated with TYP medium at the target bacterial concentration of 1 × 10^6^ CFU/ml. After 24 h incubation at 37°C, each bacterial solution was diluted 10 times, and 10 *μ*l of solution was plated on TPY solid medium by the coating method. The number of bacteria was counted after incubation for 24 h.

### 2.4. Effect of Zn-HA on Dental Enamel Cold-Light-Activated Tooth Bleaching Treatment

#### 2.4.1. Tooth Preparation

Ten sound premolars with no apparent evidence of extrinsic staining, enamel hypoplasia, caries lesions, dental fluorosis, cracks, or other defects on the teeth were collected. The teeth were ultrasonically cleaned before being sectioned into four cubic sections approximately 3-4 mm (mesiobuccal, distobuccal, mesiolingual and distolingual specimens) with a water-cooled saw. The natural tooth surfaces were serially flattened and polished using 600–3000 grit SiC paper.

#### 2.4.2. Cold-Light Bleaching Procedure

The 40 tooth blocks were randomly divided into 4 groups: groups HA, Zn-HA, deionized water (DW), and control without any treatment. The in-office bleaching protocols were performed as follows: (i) bleaching gel made of 35% hydrogen peroxide solution and silicon dioxide catalyst (BEYOND II, Beyond Technology Ltd., Centennial, CO, USA) was placed on the tooth blocks of groups HA, Zn-HA, and deionized water (DW); (ii) a cold-light source (BY-0398, Beyond Technology Ltd.) vertically above illuminated the tooth surfaces for 8 min; and (iii) the whitening agent was removed. Steps (i)–(iii) were repeated twice, and the tooth surfaces were cleaned with flowing deionized water for 1 min.

#### 2.4.3. Cycling Treatment of Tooth Specimens

Treatment solution was prepared daily for cycling with deionized water (group HA: 10 wt% slurry of HA powder; group Zn-HA: 10 wt% slurry of 8% Zn-HA powder made above; group DW: deionized water). Demineralizing solution (50 mM acetate, 2.25 mM CaCl_2_ 2H_2_O, and 1.35 mM KH_2_PO4; 130 mM KCl for pH = 5.0) and buffer solution (20 mM HEPES, 2.25 mM CaCl_2_·2H_2_O, and 1.35 mM KH_2_PO_4_; 130 mM KCl for a pH = 7.0) were prepared on the first day of cycling.

The samples were subjected to pH cycling 6 times a day for 8 days (demineralization and remineralization) in conjunction with the treatments according to the groups. Initially, each fragment was immersed in 50 ml of treatment solution for 10 minutes, then in 50 ml of demineralizing solution for 30 minutes, and finally in 50 ml of buffer solution for 10 minutes. All solutions were in constant agitation. These pH cycles/treatments were performed 6 times a day for 8 days. Between the treatment and pH cycling model, the specimens were rinsed with deionized water for 3 min. Between the daily cycling treatments, the specimens were stored in buffer solution at 37°C. After applying each phase of the pH cycling model, specimens were rinsed with deionized water for 30 seconds to prevent the cross reaction of solutions.

#### 2.4.4. XRD Analysis of Tooth Specimens

To evaluate the crystal phase and size changes in the enamel of the three groups (groups HA, Zn-HA, and deionized water (DW)) with cold-light bleaching treatment, specimens were analysed before and after the bleaching procedure using microarea XRD by the above method.

#### 2.4.5. SEM Analysis of Tooth Specimens

All tooth blocks were carefully cleaned, fixed (2.5% glutaraldehyde, 4°C, and 4 h), dehydrated, and gold-palladium sputtered for morphology analysis by SEM with resolution of 1.3 nm.

### 2.5. Statistical Analysis

All of the experiments were performed at least three times. Data are presented as the mean ± SD. All of the statistical analyses were performed using Student *t*-test using SPSS 16.0 statistical software (SPSS Inc., Chicago, IL, USA). *p* < 0.05 was considered statistically significant.

## 3. Results

### 3.1. Physicochemical Properties of Zn-HA

#### 3.1.1. XRD

**Figure 1:**
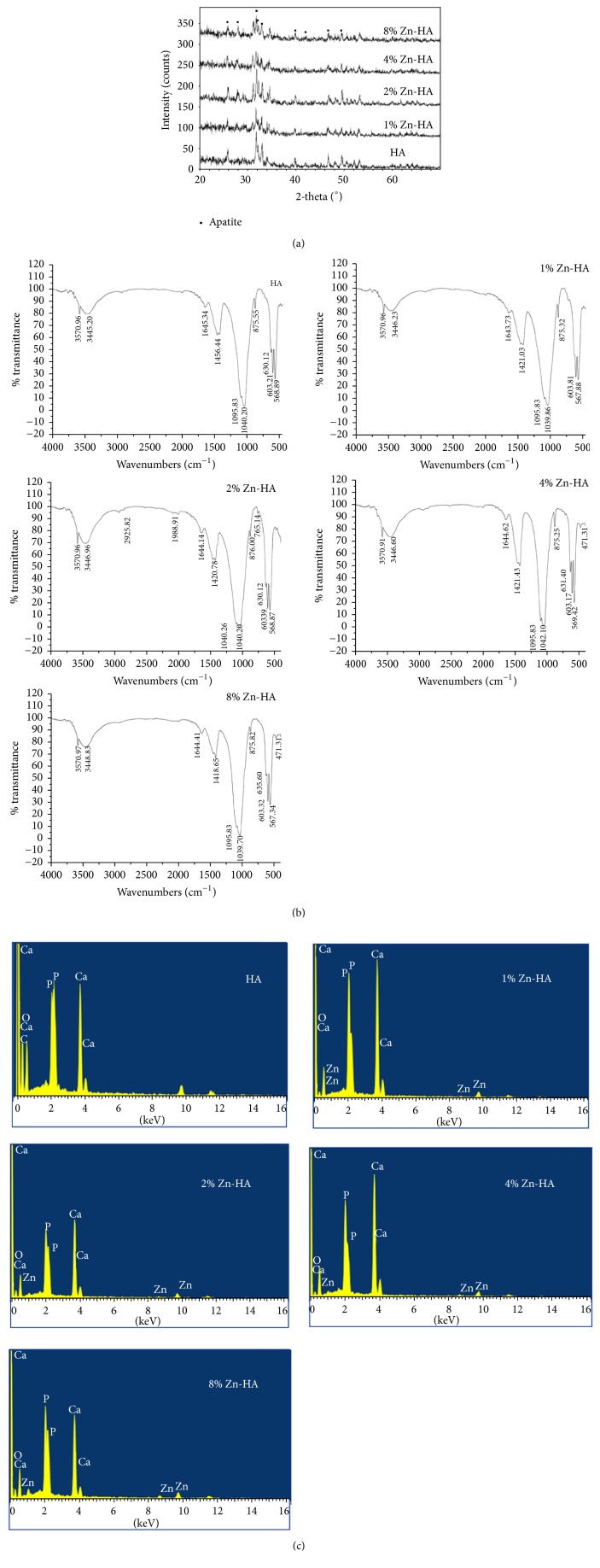
Physicochemical properties of Zn-HA (a) XRD pattern of Zn-HA. The diffraction pattern was analysed over 2*θ* range of 20°–70°. (b) The FTIR spectrum of Zn-HA. (c) Elemental chemical composition analysis using EDS.

Figure 1(a) shows the XRD pattern of Zn-HA with various zinc concentrations. The broad diffraction peaks suggested that this biomaterial was made up of nanosized crystals and low crystallinity. Furthermore, with the increasing zinc concentration, the XRD pattern was not clearly affected, as phase-pure HA was produced. The difference is considered to be caused by the constant Ca/P ratio in the synthesis. According to the cell parameter results obtained by Rietveld refinement of the XRD data (Table 1), the lattice cell parameters of Zn-HA continue to decrease with substitution of Zn^2+^ ions increasingly getting into the apatite structure, following the trend described by other works [8]. It makes sense that both the *a*- and *c*-axes decreased with Zn substitution since the size of Zn^2+^ ions (0.074 nm) is smaller than that of Ca^2+^ (0.099 nm).

#### 3.1.2. FTIR

The FTIR spectrums of Zn-HA with different Zn concentrations are compared in Figure 1(b). The absorption bands are assigned to phosphate (PO_4_^3−^) vibrations, and they were identical to the characteristics of HA, which suggests that the substitution of Zn^2+^ ions did not modify the internal structure of the HA. The v4 PO_4_^3−^ bending bands were located at 568 and 603 cm^−1^, whereas the v3 PO_4_^3−^ stretching bands were situated at approximately 1,040 and 1,095 cm^−1^. The bands located at approximately 631, 3446, and 3570 cm^−1^ are due to the OH. The bands observed at approximately 876, 1420, 1456, and 1644 cm^−1^ correspond to the CO_3_^2−^ groups in enamel.

#### 3.1.3. EDS

Figure 1(c) shows the elemental chemical composition analysis of Zn-HA using EDS, which revealed the presence of calcium (Ca_2p_) and phosphorus (P_2p_) peaks and Zn_2p3_. This result suggested that Zn^2+^ ions were structurally incorporated into the HA crystal structure by replacing the Ca^2+^ ions. EDS spectra were also obtained for analysing the surface ratios and nominal composition (Table 2).

### 3.2. Biological Assessment of Zn-HA

#### 3.2.1. Cell Proliferation

**Figure 2:**
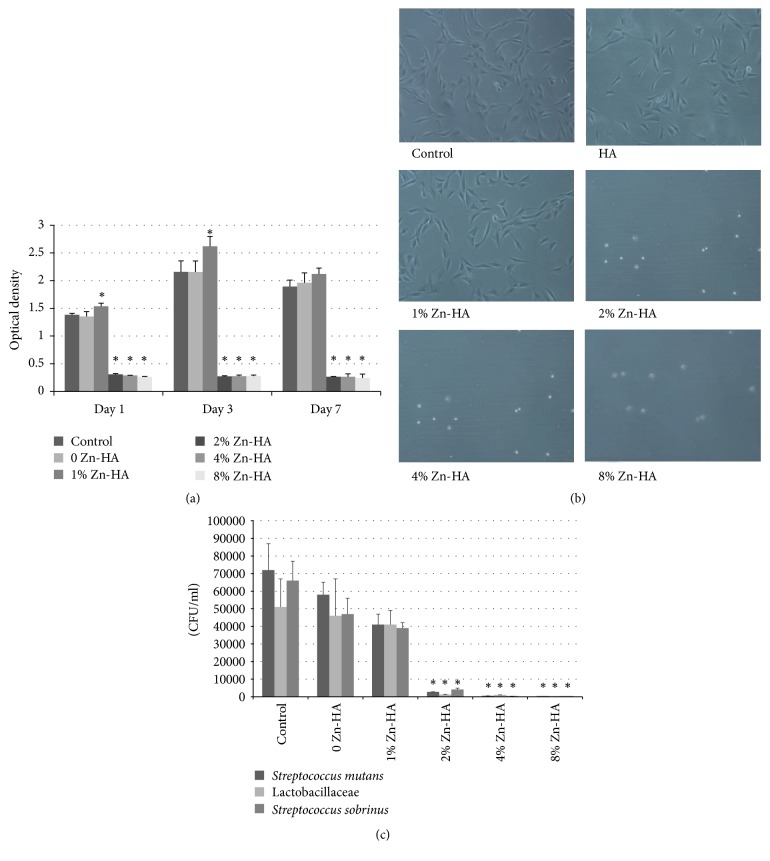
Biological assessment of Zn-HA (a) cck-8 analysis of proliferation of MC3T3 cells cultivated with Zn-HA leaching solutions for 1, 3, and 7 days. Compared with the control group, 2%, 4%, and 8% Zn-HA leaching solution treatment significantly reduced cell viability, while for 1 day and 3 days 1% Zn-HA leaching solution has an effect of promotion on the cell proliferation. (b) Morphological changes of MC3T3 cells after 24 h incubation with Zn-HA leaching solutions. (c) Antibacteria test of Zn-HA with different zinc concentration after incubation with Zn-HA solution for 24 h. Groups 2% and 4% Zn-HA and 8% Zn-HA significantly inhibited* Streptococcus mutans*, Lactobacillaceae, and* Streptococcus sobrinus* growth (*p* < 0.05). *∗* means there are statistical differences between these group and control group.

To evaluate the cytocompatibility of Zn-HA, the CCK-8 cytotoxicity assay was performed after 1, 3, and 7 days of cultivation of MC3T3-E1 cells with leaching solution from the 5 groups of Zn-HA. As displayed in Figure 2(a), a decrease in absorbance from day 1 to day 7 was recorded for groups 2% Zn-HA, 4% Zn-HA, and 8% Zn-HA (*p* < 0.05), which indicates that these three materials are toxic for osteoblasts, while 1% Zn-HA leaching solution has an effect of promotion on the cell proliferation for 1 day and 3 days (*p* < 0.05). Additionally, the OD values of groups 0 Zn-HA and 1% Zn-HA show that they have no inhibitory effect on the proliferation of osteoblasts (*p* > 0.05).

#### 3.2.2. Cell Morphology

The cytological examination of MC3T3-E1 incubated with Zn-HA leaching solutions is depicted in Figure 2(b). The morphologic observation of cell changes revealed that cells incubated with 0 Zn-HA and 1% Zn-HA leaching solutions remained polygonal and did not show obvious cell differentiation, and the cell abundance was not changed compared to the control. This suggested that Zn-HA at a zinc concentration of 1% presents no obvious cytotoxicity and could be used for clinical applications. Additionally, most cells in groups 2% Zn-HA, 4% Zn-HA, and 8% Zn-HA showed severe shrinkage to death, and the adherent phenomenon was weakened.

#### 3.2.3. Bacterial Inhibition Test

In this work, the bacterial strains* Streptococcus mutans*, Lactobacillaceae, and* Streptococcus sobrinus* were employed during the antibacterial test. Antibacterial activity was determined by counting the colony number of the bacteria after 24 h incubation with Zn-HA leaching solution.  Figure 2(c) clearly demonstrates that the Zn-HA leaching solution of groups 2% Zn-HA, 4% Zn-HA, and 8% Zn-HA inhibited growth of the antibacterials (*p* < 0.05), whereas the bacterial number of the hydroxyapatite sample without zinc exhibited no significant distinction compared with the control.

### 3.3. Assessment of Dental Enamel

#### 3.3.1. XRD Analysis

**Figure 3:**
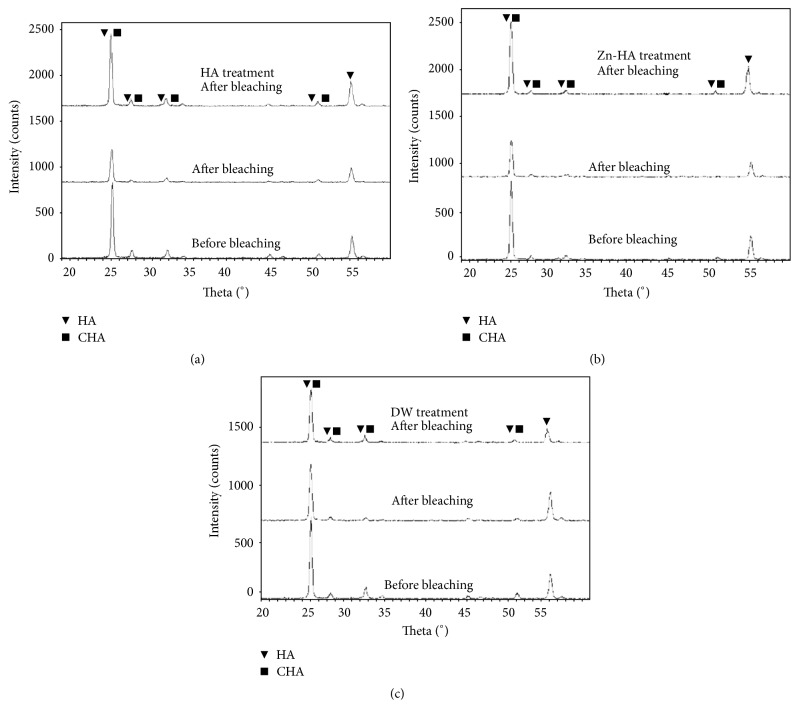
XRD patterns of dental enamel specimen before/after bleaching and pH cycling treatment. (a) Group HA. (b) Group Zn-HA. (c) Group DW. “HA” and “CHA” refer to “hydroxyapatite” and “carbonated hydroxyapatite,” respectively.

The XRD patterns of specimens from three groups (groups HA, Zn-HA, and DW) before and after cold-light bleaching treatment are displayed in Figure 3. The major phases of the specimens are hydroxyapatite (HA) and carbonated hydroxyapatite (CHA). Some peaks in the XRD pattern obviously visible before cold-light bleaching are revealed to be weaker or even have disappeared in the posttreatment pattern. After 8 days of pH cycling, the diffraction peaks of groups HA and Zn-HA are restored to those before cold-light bleaching, but the peaks of group DW are as weak as those observed immediately following cold-light bleaching. Compared with group DW, the diffraction peaks of groups HA and Zn-HA after pH cycling significantly increase.  Table 3 shows the changes of crystal size before/after the cold-light bleaching treatment and after pH cycling treatment. Generally, the crystal size of all groups significantly decreased after cold-light bleaching. The crystallinity of groups HA and Zn-HA is mainly restored after pH cycling treatment. There is no statistical significance (*p* > 0.05) between the group HA and group Zn-HA XRD data.

#### 3.3.2. Scanning Electron Microscopy of Enamel Surfaces

**Figure 4:**
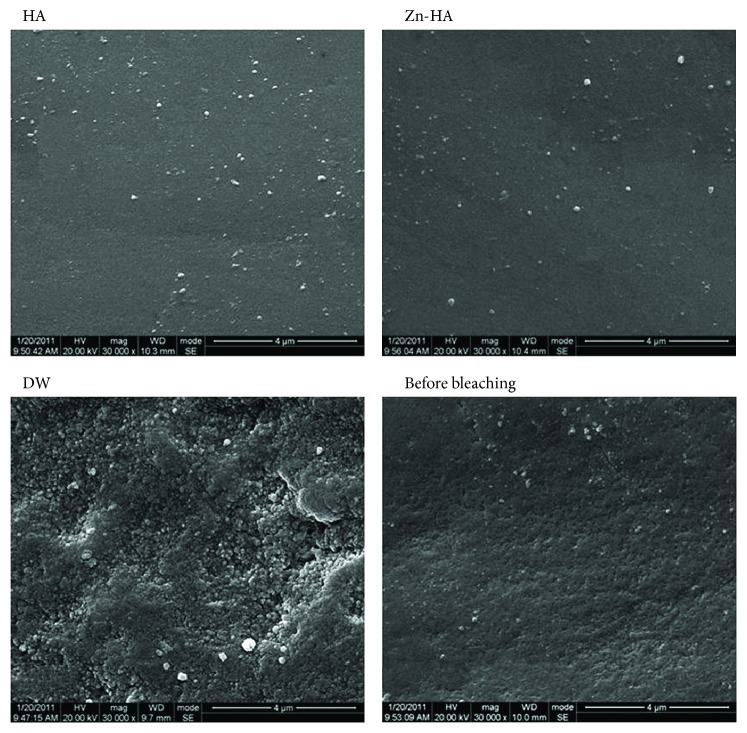
Scanning electron microscopy (SEM) micrograph of dental enamel surface after pH cycling treatment. The images were obtained at 30000x magnification.

Figure 4 shows morphological analysis in the central area of the enamel surface before and after the bleaching treatment. The surface morphology appeared smooth before bleaching, and, for all groups of tooth blocks, a rough surface was observed after bleaching treatment. Moreover, a clear spherical enamel crystal structure can be identified, indicating the demineralization effects on the tooth enamel surface from the cold-light bleaching treatment. After pH cycling treatment, the enamel surfaces of group HA and Zn-HA were observed to be smooth again and were basically restored to the level before bleaching.

## 4. Discussion

Zinc ion-substituted hydroxyapatite particles were obtained by the wet chemical method as detected from XRD, FTIR, and EDS results. There still has been some controversy regarding the forms of zinc present in hydroxyapatite, and the main two viewpoints are as follows: (1) zinc ions adsorb on the surface of the HA crystal, which causes the X-ray diffraction pattern peaks to become wider and lower, as the presence of zinc ions affects the growing process of the crystals and the crystallinity is decreased; and (2) when Zn^2+^ is incorporated into the lattice structure of HA, the central Ca atom is replaced with Zn [9]. The cell parameters and size are reduced because Zn^2+^ ions have a smaller ionic radius of 0.74 Å compared to Ca^2+^ (0.99 Å), as shown in Figure 1 and Table 1. In this study, the XRD pattern figures indicate that zinc ions exist in both forms. The FTIR spectra in Figure 2 reveal a decrease in relative peak intensity at 568 and 603 cm^−1^ for Zn-doped HA compared to undoped HA, also reflecting a relative decrease in crystallinity with addition of dopants into the HA nanocrystal.

The biocompatibility of Zn-HA is an important criterion for its clinical application. We evaluated the biocompatibility of different concentrations of zinc-substituted HA using the CCK-8 assay. A statistically significant decrease in cell viability was observed for 2% Zn-HA, 4% Zn-HA, and 8% Zn-HA after a 24 h incubation. Sogo et al. reported that release of zinc from zinc oxide caused cytotoxicity when the zinc content was higher than 1.20 wt% [10]. In this experiment, the Zn/(Ca+ Zn) ratios are 1%, 2%, 4%, and 8%, equaling 0.61 wt%, 1.29 wt%, 2.57 wt%, and 5.11 wt%, respectively, for the quality fraction of samples, which is consistent with previous studies. The cell proliferation experiment revealed that the absorbance of MC3T3-E1 cells incubated with leaching solution originated from 1% Zn-HA increased, illustrating that zinc may positively affect osteoblast proliferation. Previous studies have reported that Zn-HA has favourable effects on reducing inflammation and improving monocyte chemotaxis [11]. Additionally, zinc ion promotes the anabolic effects of insulin-like growth factor-I (IGF-I) in MC3T3 osteoblastic cells by increasing both the alkaline phosphatase activity and protein concentration [8, 12], due to the fact that the zinc binding protein metallothionein plays an important role in the regulation of cell cycle and differentiation [13]. Not only can zinc promote osteoblast proliferation and differentiation, but it has also shown an inhibitory effect on osteoclastic bone resorption [14].

*S. mutans*, Lactobacillaceae, and* S. sobrinus *are common in dental caries, and their metabolites improve enamel demineralization. We proved in the antibacterial test that Zn-HA exhibits significant antibacterial activity against* S. mutans*, Lactobacillaceae, and* S. sobrinus*, and this inhibitory effect was positively correlated with the zinc ion concentration. The antibacterial mechanism consists of zinc inhibiting the enzyme metabolism of caries bacteria and destroying plaque formation [15].

Enamel demineralization is a widespread problem during cold-light bleaching treatment, and it produces a rougher and irregular surface that facilitates plaque and bacterial retention and might translate into caries disease. Jiang et al. used the combination of hydroxyapatite (HA) powder and 30% hydrogen peroxide in a mixture as a tooth bleaching product, and it was effective at tooth whitening [5]. Furthermore, it significantly reduced the microhardness loss of enamel caused by 30% hydrogen peroxide. In this study, it is worth noting that the enamel surface morphology was made smooth again by utilizing HA-Zn in pH cycling treatment, thus indicating its possible function of reducing demineralization. The weakly alkaline HA and Zn-HA could reduce demineralization by counteracting the effect of acid, and nanosized HA and Zn-HA can block the effect of acid attack on enamel by adhering to the enamel surface and forming a protective layer for the underlying enamel. In addition, their high solubility greatly improves the concentration of Ca^2+^ and PO_4_^3−^ ions and promotes the formation of HA crystallization.

## 5. Conclusions

Zn-HA and HA solutions significantly reduce the microhardness loss of enamel caused by cold-light bleaching and keep the enamel surface morphology almost unchanged. Zn-HA shows superior ability to inhibit plaque formation and acid production, and, therefore, Zn-HA is the more advantageous choice as compared to HA for enamel remineralization in dental prosthetic restoration after cold-light bleaching.

## Figures and Tables

**Table 1:** Lattice cell parameters of Zn-HA obtained from Rietveld refinement.

Group	*a* (nm)	2*θ*	*c* (nm)	2*θ*
0 Zn-HA	9.4341	32.860°	6.8926	25.830°
1% Zn-HA	9.4196	32.911°	6.8926	25.830°
2% Zn-HA	9.3908	33.015°	6.8790	25.882°
4% Zn-HA	9.4047	32.965°	6.8926	25.830°
8% Zn-HA	9.4168	32.922°	6.8872	25.851°

**Table 2:** EDS atomic ratios calculated from peaks of references and samples.

	Zn/(Ca + Zn)	(Zn + Ca)/(P + C)
0 n-HA	0	1.6704
1% Zn-HA	0.0105	1.6591
2% Zn-HA	0.0218	1.6678
4% Zn-HA	0.0388	1.6708
8% Zn-HA	0.0837	1.6801

**Table 3:** The mean (and SD) of the changes in crystal size (nm) and crystallinity.

	Post-Cold-light bleaching vs pre-cold-light bleaching	Post-pH cycling versus pre-pH cycling	Post-pH cycling treatment versus pre-cold-light bleaching
HA	−2.90 (2.17)^*∗*^	2.75 (1.83)	−0.15 (0.13)
Zn-HA	−2.85 (2.31)^*∗*^	2.74 (1.97)	−0.12 (0.21)
DW	−2.87 (1.66)^*∗*^	0.12 (0.17)	−2.75 (1.97)^*∗*^

^*∗*^Student's *t*-test indicates a statistically significant difference (*p* < 0.05).
